# Alfred Binet and the Concept of Heterogeneous Orders^†^

**DOI:** 10.3389/fpsyg.2012.00261

**Published:** 2012-08-15

**Authors:** Joel Michell

**Affiliations:** ^1^The University of Sydney, School of PsychologySydney, NSW, Australia

**Keywords:** homogeneity, Intelligence Tests, measurement, order, qualitative, quantitative

## Abstract

In a comment, hitherto unremarked upon, Alfred Binet, well known for constructing the first intelligence scale, claimed that his scale did not measure intelligence, but only enabled classification with respect to a hierarchy of intellectual qualities. Attempting to understand the reasoning behind this comment leads to an historical excursion, beginning with the ancient mathematician, Euclid and ending with the modern French philosopher, Henri Bergson. As Euclid explained (Heath, 1908), magnitudes constituting a given quantitative attribute are all of the same kind (i.e., homogeneous), but his criterion covered only extensive magnitudes. Duns Scotus (Cross, 1998) included intensive magnitudes by considering differences, which raised the possibility (later considered by Sutherland, 2004) of ordered attributes with heterogeneous differences between degrees (“heterogeneous orders”). Of necessity, such attributes are non-measurable. Subsequently, this became a basis for the “quantity objection” to psychological measurement, as developed first by Tannery (1875a,b) and then by Bergson (1889). It follows that for attributes investigated in science, there are three structural possibilities: (1) *classificatory attributes* (with heterogeneous differences between categories); (2) *heterogeneous orders* (with heterogeneous differences between degrees); and (3) *quantitative attributes* (with thoroughly homogeneous differences between magnitudes). Measurement is possible only with attributes of kind (3) and, as far as we know, psychological attributes are exclusively of kinds (1) or (2). However, contrary to the known facts, psychometricians, for their own special reasons insist that test scores provide measurements.

This scale properly speaking does not permit the measure of the intelligence, because intellectual qualities are not superposable, and therefore cannot be measured as linear surfaces are measured, but are on the contrary, a classification, a hierarchy among diverse intelligences; and for the necessities of practice this classification is equivalent to a measure. (Binet and Simon, 1980, pp. 40–41)

Anyone who knows what scientific measurement^1^ is, also knows that psychometric testing is not measurement in the same sense as that term is used in physical science to describe assessment of quantitative attributes like distance, mass, or temperature. While some psychometricians realize this, most do not and they typically regard tests as instruments of scientific measurement. Indeed, some make a special point of stressing their credentials (for example, Bond and Fox, 2007) for allegedly achieving measurement. Also, it is not generally known that the attitude of ignoring the logic of scientific measurement was present at the birth of psychometrics. This attitude was not something that only emerged later in the history of this discipline when its credentials were questioned. From the very beginning there was a mindset that advocated only one possibility: tests measure.

There is a poignant vignette in the history of testing, one hitherto unexamined, which illustrates this point and, at the same time, draws attention to an important, but long neglected concept. This vignette involves the Frenchman, Alfred Binet^2^ and the American, Lewis Terman^3^. As is well known, Binet (with Simon) constructed the first “intelligence scale^4^,” which Terman adapted for American use, eventually producing the “Stanford–Binet scale^5^.” Less well known is that Binet^6^ thought this about his test:

This scale properly speaking does not permit the measure of the intelligence, because intellectual qualities are not superposable, and therefore cannot be measured as linear surfaces are measured, but are on the contrary, a classification, a hierarchy among diverse intelligences. (Binet and Simon, 1980, p. 40)

When Terman read this he underlined the phrase, “a hierarchy among diverse intelligences,” and, venting incomprehension, scribbled one word and a punctuation mark: “meaning^7^”? Despite Binet’s phrase being pregnant with meaning, for Terman, this meaning fell stillborn and, as an objection to testing, was never raised again.

What did Binet mean by the remark, “intellectual qualities … are a classification, a hierarchy among diverse intelligences”? That this has gone undiscussed is odd given that the preceding remark, viz., “intellectual qualities are not superposable, and therefore cannot be measured as linear surfaces are measured” has been noted more than once (see for example Gould, 1981, p. 151; Nash, 1987, p. 76; Michell, 1999, p. 94). At the time, measurement was thought to depend upon equality between magnitudes, which in the case of linear surfaces may be established by superposing, say, rigid straight rods, thus identifying a set all equal to a given unit. This allows the length of another object to be assessed by counting equal units along its extent. So, this first part of Binet’s remark raises the objection that measurement depends upon addition of units, which in turn presupposes that the attribute involved possesses additive structure. I have already drawn attention to this presupposition (see for example, Michell, 1997, 1999, 2000, 2001, 2002, 2003, 2004, 2007, 2008, 2009) so let me relate what Binet meant by the second part of his remark.

Before Binet found fame, he was an experimental psychologist (see Wolf, 1973; Nicolas and Ferrand, 2002; for an account of Binet’s research and difficulties). Much of his research was done as director (without remuneration) of the laboratory of physiological psychology at the Sorbonne, but Binet was repeatedly thwarted in his attempts to gain a teaching position in psychology at that university. Psychology was then seen as an area of philosophy and a teaching position was denied him because he had no formal training in that discipline. Attempting to rectify this, he wrote articles and a book on the mind-body problem (Binet, 1905), but all to no avail. However, he was very well versed in philosophical issues relevant to psychological research and, presumably, those relating to the controversies then engulfing psychophysical measurement.

One of these was due initially to a French mathematician and later expanded by a French philosopher (see Titchener, 1905; Heidelberger, 2004). The mathematician, Jules Tannery^8^, criticized Fechner’s (1860) claim to have devised methods enabling measurement of intensity of sensations. Fechner had been trained as a physicist and when he said that he could measure sensations he used the term “measure” in the same sense as it is used in quantitative physics. In physics, the measure of some magnitude is its ratio to whatever unit is being employed and Tannery’s argument was that sensation intensities are not measurable because they lack the special kind of homogeneity necessary for measurement^9^. The magnitudes of any given quantitative attribute are all homogeneous. That is, as the magnitudes of some quantity, such as length, increase by the repeated addition of the same unit, the meaning of the unit does not alter. For example, one meter added to ten is the same length as when added to one hundred. That is, any two distinct lengths only ever differ quantitatively, never qualitatively. This is part of what it means for length to be quantitative. However, comparing, say, sensations of heat, Tannery claimed that we find, as these sensations become more intense, they differ qualitatively from one another, for example, at one extreme involving pain, at the other not. Tannery’s point is that the sensation experienced is not simply one of heat, but is a complex ensemble of various other feelings as well, such as pain or pleasure, and it is the hierarchy of these ensembles that, while ordered, contains qualitative differences between degrees. Hence, while he agreed that sensations within a given modality could be ordered according to intensity, he thought that they could not be measured because the relevant attribute (i.e., the series of sensations) possesses heterogeneous differences between its degrees and, so, cannot be quantitative^10^.

Leaving aside whether he was right in his theory of sensations, let us call any ordered attribute with heterogeneous differences between its degrees, a *heterogeneous order*. Implicit in Tannery’s objection is the claim that there are three different sorts of attributes in the world: classifications, such as, for example, the classification of people according to nationality; heterogeneous orders, such as Tannery was claiming sensation intensities to be; and quantitative attributes, such as length, temperature, etc. As I will argue, Tannery was right about this at least. Only quantitative attributes can be measured because only they possess the necessary kind of homogeneity. This is not to suggest, however, that classifications and heterogeneous orders cannot be investigated scientifically, only that when investigated, they must be assessed in other ways.

The philosopher, Henri Bergson^11^, while agreeing that sensation intensities are not quantitative, muddied the waters by insisting that if an attribute is ordered, if it admits relations of “more” and “less,” then it must also be quantitative. He thought that if the degrees of an attribute are ordered, this is only because they stand in relations of inclusion to one another, greater degrees always including all lesser. Because for him the model of inclusion was spatial inclusion – a quantitative relation – he concluded that order always entails quantity^12^. Consequently, he thought, each sensation is a pure quality, neither greater nor less than any other and all we can do with sensations is classify them. According to Bergson (1889), the conviction that they are ordered is really an illusion caused by extraneous accompaniments of the circumstances of their occurrence. For Bergson then, there are only two kinds of attributes: classifications, and quantitative attributes^13^.

Although I have no direct evidence, I conclude that Binet was aware of Bergson’s writings on psychophysics and since Bergson refers to him, of Tannery’s as well. My reasons for this assessment are as follows: psychophysics was then the most important area of experimental psychology^14^ and, initially, “Binet’s goal was to be recognized as the leader of experimental psychology in France” (Nicolas and Ferrand, 2002, p. 265); Bergson’s critique of psychophysics was well known in France and is said to be the main reason why experimental psychology got off to such a slow start there (Nicolas and Murray, 1999); and, furthermore, Binet was well aware of Bergson and his work, having referred to Bergson in other writings^15^.

Interpreted in this light, Binet’s remark may be understood as drawing upon both Tannery and Bergson. Of course, Binet was not, like Tannery and Bergson, discussing sensation intensities but was discussing the cognitive states sustaining performance on intellectual tasks: “intellectual qualities,” as he called them. At first, Binet, like Bergson, seemed to recognize only two possibilities: classification and measurement, with intellectual qualities only amenable to the former. Consistent with this, in an earlier paper (Binet, 1898), he had suggested that higher mental functions, like “acuteness of intelligence” could only be classified, not measured, again making his point as if these were the only two possibilities^16^. But then he added that intellectual qualities form a hierarchy – that is, an order – among diverse intelligences. Now, had he been following Bergson’s line, he would have concluded either that intellectual qualities are measurable (because Bergson thought that order entails quantity) or that the ordering of intellectual qualities that his scale achieved was illusory, which he clearly did not believe. It is clear from the discussion that follows that Binet thought that ordered degrees of intelligence were real and could be assessed. Hence, I conclude that he did not follow Bergson’s line. As the phrase “diverse intelligences” indicates, he seems to have thought that the reason intelligence cannot be measured is because the cognitive states underlying test performance are not quantitatively homogeneous, but differ from one another in heterogeneous ways. Thus, they constitute what I am calling a heterogeneous order. It is this “diversity,” this heterogeneity, which Binet thought rules out measurement.

But why was it thought that heterogeneity rules out measurement? It is because measurement of quantitative attributes requires that they possess the special property of *quantitative homogeneity*. Around 300 BC, Euclid, compiled his *Elements*. Book V touched on the topic of measurement (see Heath, 1908). Euclid noted that the magnitudes^17^ of any given quantitative attribute, such as length, are *magnitudes of the same kind*, that is, *homogeneous*. For example, all lengths, say, the length of this room and the length of your shoe, are magnitudes of the same kind. And we can tell this, thought Euclid^18^, because if we take any length, like the length of your shoe, and multiply it some finite number of times, it will exceed any other length, like, say, the length of this room. This tells us that these two lengths are homogeneous because it means that the length of this room falls between two lengths in the series of multiples of your shoe length. This series must be homogeneous because it contains multiples of exactly the same length, viz., length of your shoe, and, so, if the length of this room can fall between items within this series, it must be homogeneous with the lengths constituting it.

Now, this criterion of homogeneity is fine, but limited. It works with *extensive* attributes, that is, quantitative attributes like length, where multiples can be constructed, but it does not work with *intensive* attributes, like temperature. This did not matter in ancient times because then only extensive attributes were measured, like length, area, volume, plane angle, weight, and time. Ancient philosophers, like Plato^19^ and Aristotle^20^ could only speculate that other attributes, like say, pleasure, or temperature might be quantitative.

Of course, it did not require measurement of intensive attributes to wonder about their homogeneity. From the thirteenth century, scholars became intrigued by the fact that certain qualities, like charity or whiteness, occur in different degrees^21^, and that these degrees are subject to change. That is, one person might possess less charity than a second 1 day, but later, the first might come to have more charity than the second; or one shirt might be whiter than another 1 day, but not as white the next. The puzzle was how to think about different degrees of a given quality and how to conceptualize change from one degree to another (see Crombie, 1994).

There are only two possibilities. I call them the *qualitative* and the *quantitative*. On the qualitative view, each distinct degree of a quality, such as whiteness, is *qualitatively different* from the rest. So what we would have with the range of shades we call degrees of whiteness would be a series of discrete grades approaching pure whiteness, but each differing from the other in some qualitative way, say, due to the presence of some different kind of impurity mixed in with the white. By contrast, on the quantitative view, each different degree of some quality is *quantitatively different* to each other, so that what we have with a quality such as whiteness would be a continuous series of shades approaching pure whiteness.

This problem was made the harder because medieval philosophers revered Aristotle who taught that qualities and quantities are different categories of existence and that they exclude one another. In particular, Aristotle had taught both that “Quantity does not, it appears, admit of variation of degree^22^” and “Qualities admit variation of degree^23^.” What Aristotle meant is that there are no degrees of any quantitative attribute, such as *being four feet in length*: an object either *is* or it *is not* four feet. On the other hand, qualities, such as *being white*, admit degrees. That is, one thing may be whiter than another. Furthermore, it was widely believed, especially in the early middle ages, that qualities were more important than quantities in understanding how the physical world worked (Crombie, 1994). So, medieval philosophers initially endorsed the qualitative option. However, the British philosopher, John Duns Scotus^24^, convinced them that the quantitative option was superior, especially for explaining change. As Richard Cross explains Scotus’ position: for any quality, *Q*, “a change from one degree *D* to another *E* is explained by the addition and subtraction of (homogeneous) parts of *Q*” (Cross, 1998, p. 186). When Scotus’ solution caught on, the momentum of the ensuing conceptual revolution was unstoppable. From the fourteenth century, philosophers conceptualized all degrees of qualities as if measurable quantities (see for example Pedersen, 1974; Lindberg, 1992; Grant, 1996). As expressed by the medieval French scholar, Nicole Oresme, “the measure of intensities (of qualities) can be fittingly imagined as the measure of lines” (Clagget, 1968, p. 167). From then onward, this conceptualization of qualities became a permanent feature of scientific thought and it became axiomatic in psychology from the second half of the nineteenth century. It is echoed in slogans such as Thorndike’s *credo*, which still reverberates through the discipline (Michell, 2005): “Whatever exists at all exists in some amount. To know it thoroughly involves knowing its quantity as well as its quality” (Thorndike, 1918, p. 16).

How did those following Scotus understand quantitative homogeneity in relation to the degrees constituting a specific quality? His treatment forced them to focus upon *differences between degrees*. If degrees of some quality are quantitative, then differences between degrees, also, must only differ quantitatively, not qualitatively. This defines quantitative homogeneity: not only are all magnitudes of a given quantity magnitudes of the same kind; and not only are differences between all pairs of magnitudes likewise of the same kind; but also, and this is the crucial point, these differences cannot differ from one another in any qualitative way. Different magnitudes of the same quantitative attribute never differ qualitatively.

This position does not rule out the possibility that objects possessing different degrees of some attribute might differ qualitatively from one another. For example, as temperature increases, ice turns to water, which in turn turns to steam and these different states of water differ qualitatively. But this does not mean that temperature differences likewise differ qualitatively. Objects must be distinguished from attributes and whether any substance is solid, liquid, or gas depends upon other properties it possesses, not just upon its temperature, as is clear from the fact that different substances liquefy or vaporize at quite different temperatures. However, the quantitative attribute of temperature, itself, which is now understood in physics as a property of a body’s internal energy, is such that differences between its magnitudes never differ qualitatively.

Conversely, the degrees of a mere quality, if such exist, would differ from one another only qualitatively. The degrees of such a quality would still all be homogeneous, in the sense that they would all be degrees of the same quality, and differences between the degrees would also be homogeneous in the sense that they would all be differences between degrees of the same quality. However, these differences between degrees would also be qualitatively different and, hence, heterogeneous. This may sound contradictory, but it is not. We encounter collections of things that are both homogeneous and heterogeneous. For example, a collection of people is always homogeneous in the sense that it is a collection of people. However, it may also be heterogeneous in the sense that it may contain people of different kinds, say, males, and females. The important distinction here is between collections that are thoroughly homogeneous, such as the magnitudes of a quantity and collections that are both homogeneous and heterogeneous, such as degrees of a quality. Quantitative homogeneity is pure. Non-quantitative homogeneity is impure.

So we can see that from a logical point of view, Tannery was right, three different kinds of attributes are possible: *classifications*, where there will be heterogeneous differences between classes, but the classes are not ordered; *heterogeneous orders*, which admit qualitative differences between degrees and, so, the degrees are not measurable; and *quantitative attributes*, which admit no heterogeneity in differences between magnitudes and, so, are measurable.

Heterogeneous orders might be logically possible, but do they ever actually exist? The medieval philosophers never asked this, so seduced were they by the perceived merits of Scotus’ suggestion. Their neglect had one positive outcome: an intellectual climate conducive to the Scientific Revolution, in so far as attempts to measure intensive quantitative attributes, like velocity, density, and temperature were concerned. But, it also had a negative side: false expectations regarding ordered attributes generally, for it was presumed that in principle, every ordered attribute must be measurable.

*A priori*, this is highly implausible. There are indefinitely many concepts to which we apply degree words (see Bolinger, 1972; Engel, 1989): for example, arguments may be more or less rigorous; procedures, more or less efficient; sketches, more or less life-like; songs, more or less romantic; prisons, more or less secure; and so on. Is it safe to conclude, without further ado, that in each such case, the relevant ordered attribute is quantitative and, therefore, in principle measurable? If we were to look closely at, say, different degrees of security in prisons, might we not find that it is qualitatively different factors that constitute increasing levels of security? At least, we cannot rule out this possibility *a priori*.

Over the centuries, a range of views emerged, with, at one extreme, some, like Thomas Reid, berating Francis Hutcheson, for “applying measures to things that properly have not quantity^25^” and, at the other extreme, others, such as Thorndike chanting his *credo*. Reid assumed what Thorndike denied, that not every ordered attribute is quantitative. Few showed Reid’s perspicacity and most followed the quantitative path, although some, such as the philosopher, Curt Ducasse, moderated it by claiming that “the non-measurability of something that observably admits of more and less is never known to be an intrinsic character of it^26^.” However, this latter view leaves the gate to the quantitative path perpetually ajar, by denying that non-measurability could be an intrinsic characteristic of ordered attributes. It means that the issue can only ever be decided in one direction (i.e., by establishing that an attribute is quantitative) and never in the other (i.e., it cannot be established that an ordered attribute is not quantitative). Were this the case, psychometricians could build their quantitative castles in the air, safe in the conviction that they can never be shown to be wrong because, on this view, one can never validly conclude that an ordered attribute is non-quantitative.

However, Reid is right and, as a matter of fact, the German philosopher, Immanuel Kant, settled the matter otherwise over a century before psychometrics was born, but his discussion is still not well known in psychology, at least^27^. Kant noted that within a series of concepts ordered according to specificity (such as, for example, the concepts of *human*, *primate*, *mammal*, *vertebrate*, *animal*), the differences between succeeding concepts, while homogeneous (i.e., they are all differences between living things) are also heterogeneous (i.e., e.g., what distinguishes humans from the rest of the primates is not the same kind of thing as distinguishes primates from other mammals, and so on). This shows that some hierarchies are heterogeneous orders^28^.

Furthermore, Kant showed why heterogeneity rules out measurability. For example, the concept of being a human *includes* that of being a primate, that of being a primate *includes* that of being a mammal, and so on. It is the relation of *conceptual inclusion* that is the basis for order in this case. However, consider the difference between humans and other primates and the difference between primates and other mammals. No such relation of inclusion exists between these differences and, so, they are intrinsically unordered. But if an order is to be quantitative, then differences between its degrees must be intrinsically ordered^29^. Ducasse was wrong: an ordered attribute is intrinsically non-measurable if the differences between its degrees are heterogeneous because then such differences are not equal to, greater than, or less than one another. Thus, non-measurability can be an intrinsic feature of an ordered attribute.

Those who nonetheless still insist, albeit wrong-headedly, that every ordered attribute is quantitative confuse *differences between degrees* of an ordered attribute with *quantitative distances* between magnitudes of a quantitative attribute. The British philosopher, David Hume, warned, “any great *difference* in the degrees of any quality is called a *distance* by a common metaphor” because “the ideas of distance and difference are … connected together” and “connected ideas are readily taken for each other” (Hume, 1888, p. 393). In other words, Hume was saying, this confusion comes about because of a cognitive illusion, viz., taking distance as a metaphor for difference. Psychologists have applied all of their ingenuity to the finding of ways whereby this illusion might be exploited. For example, psychometricians who favor item response theory (IRT) models do this by presuming certain responses to test items to be “errors” and then treating features of these “errors” as an index of the magnitude of the distances that they believe exist between degrees of ability. However, without that presumption, it is not clear that these putative distances are any more than qualitative differences and that psychometricians are merely exploiting the illusion Hume drew attention to.

While Kant showed that heterogeneous orders exist and revealed why they cannot be quantitative, he did not bring out the scientific importance of the distinction between quantitative attributes and heterogeneous orders. Quantitative attributes stand in regular quantitative relationships with one another, such as, area = length × breadth. This is made possible by the pure homogeneity of their magnitudes. For example, because there is no qualitative difference between different lengths, the relationship between length and other attributes, such as area, does not vary across the range of lengths. However, because the degrees of a heterogeneous order differ qualitatively from one another, different causal laws will apply to different degrees of the same attribute. Any science dealing with heterogeneous orders will be much more complex than quantitative sciences, such as physics. Attempting to quantify heterogeneous orders treats them in a way that belies their complexity and, thereby, falsifies our understanding of them.

This is the background to Binet’s remark that the attributes underlying test performance are not measurable, but are hierarchies among diverse intelligences. Was he right? Consider, for example, any unidimensional test of ability – say, a test of mathematical ability. It consists of a series of test items of increasing difficulty such that at each level of difficulty, the cognitive resources required to pass an item differ from those above or below it in qualitatively different ways. That is, if three items, *x*, *y*, and *z* are unidimensional (that is, all assess the same ability) and of increasing difficulty, then the difference between *x* and *y* in terms of cognitive resources required for a correct response cannot be the same as those between *y* and *z*, and so on for all such pairs. Hence, the attribute assessed by the test, which is, of course, the series of cognitive states determining different levels of test performance, is a hierarchy with heterogeneous differences between degrees. As such it is intrinsically non-quantitative. That is, Binet was right about mental abilities, in so far as their character can be inferred from the test items used in assessment: they are non-measurable attributes.

In the most clear cut case, the cognitive resources needed to pass an item at any level of difficulty subsume those needed to pass all easier items. That is, the cognitive resources constituting any degree of ability stand in relations of inclusion to all lesser degrees. Disregarding performance errors, this kind of structure in degrees of ability would sustain a Guttman scale and no doubt, given performance errors, it could produce response patterns fitting quantitative psychometric models, such as IRT models. That is, it is possible that attributes that psychometricians aspire to measure are heterogeneous orders, that is, non-measurable attributes, and this fact is not incompatible with observing statistical fit to IRT models^30^.

So, what does this tale from the archives reveal? Twenty-two years ago, discussing measurement in psychology, I wrote, “The mistake of the psychologists was to be more interested in the pursuit of their quantitative program than in the pursuit of the underlying facts” (Michell, 1990, p. 20). This tale reveals that the pursuit of a quantitative program in preference to investigation of the facts can be dated from the birth of psychometrics. This tale describes the moment when the original presumption was made that tainted everything after it. From here on, the history of psychometrics became the history of rationalizations for measurement: probabilistic, quantitative IRT models being the latest. These models contain a common feature: they presume that the attributes tests assess are all continuous, quantitative attributes. There is no evidence, independent of these models, however, supporting this presumption. Indeed, in so far as the attribute assessed by any test is constituted by the hierarchy of cognitive states sufficient for correct responses to its items, it is a heterogeneous order, not a quantitative structure. Binet, more than a century ago, alluded to this difficulty. Not only did an uncomprehending Terman turn away, but also interestingly, Binet, himself, commented that “for the necessities of practice” the classification that his test provided “is equivalent to a measure” (Binet and Simon, 1980, pp. 40–41).

In the same collection, in a paper on the 1908 version of his scale (Binet and Simon, 1908), Binet is translated as saying,

“The Measurement of Intelligence” is, perhaps the most oft repeated expression in psychology during these last few years. Some psychologists affirm that intelligence can be measured; others declare that it is impossible to measure intelligence. But there are still others, better informed, who ignore these theoretical discussions and apply themselves to the actual solving of the problem. (Binet and Simon, 1980, p. 182)

What are we to make of Binet’s apparent equivocation about whether his scale provides a measure of intelligence? He knew that his scale did not measure intelligence and yet thought that for practical purposes it was equivalent to a measure. On this basis, Nash (1987) accuses him of “intellectual bad faith” but I think that another interpretation is more likely: in drawing our attention to the distinction, I believe Binet was merely cocking his snoot at his *bête noire*, Henri Bergson. Bergson did not think that psychophysical measurement was possible and, as Binet realized, Bergson’s argument applies with equal force to Binet’s intelligence scale. As far as Binet was concerned, however, this purely philosophical objection had no value alongside the practical achievement wrought by his intelligence scale because his scale, he thought, enables us to do all that we could ask of an actual measurement device, were we in possession of one. Hence, he seems to have thought, let us be done with it and call it equivalent to a measure of intelligence. Binet’s career aspirations had been cruelled by Bergson on the grounds that Binet was philosophically unqualified to be a professor of psychology. So both Binet and his scale were in the same boat: philosophically unqualified. But just as Binet thought his scale could do all that might be asked of it without those qualifications, so he clearly thought he also was worthy of the position denied him.

Even today, it remains true that most of the practical decisions made on the basis of psychological test scores ask nothing more than that those scores order people on the attributes assessed. So, to that extent, Binet was correct. However, to take the further step, and assert that such scores are equivalent to a measure, is to license exactly the sort of confused thinking that characterizes modern psychology. Less than a decade later, an advocate of Binet’s tests, Margaret Drummond wrote, “The ideal that Binet set himself was the formation of a scale which should measure intelligence in something the same way as the foot-rule measures height” (Drummond, 1914, p. 147). This confusion was all grist to the psychologists’ mill as they sought to project the image of their discipline as a quantitative science and to market their tests as instruments of scientific measurement.

However, it might be asked, what difference would it make if, as I have argued, the kinds of attributes psychometricians aspire to measure are not quantitative? After all, they could still be assessed with respect to order and is there such a huge difference between ordinal and interval scales? One of the defects of Stevens’s well known classification of “scales of measurement” (Stevens, 1946) is that in assimilating classifications (“nominal scales” in Stevens’s terms) and orderings (“ordinal scales”) into his concept of measurement, the conceptual difference between the qualitative methods of classifying and ordering and the quantitative method of measurement is obscured. The simplest way to see this difference is to note the fact that in “nominal” and “ordinal scaling” the use of numerals is optional because all of the information contained in such “scales” can be expressed non-numerically. For example, the classes comprising a classification can be given non-numerical names and the categories constituting an ordering can be designated using terms from any ordered series, such as letters of the alphabet. On the other hand, in “interval” and “ratio” scaling, number is necessarily implicated because the information such scales contain is intrinsically numerical. This is why measurement is a *quantitative* method and classification and ordering are not quantitative but merely *qualitative* methods. Noting this, further differences would follow for the context of psychological testing were the relevant psychological attributes heterogeneous orders.

First, it would mean that the phenomena of intelligence, abilities, personality traits, and social attitudes are not quantitative phenomena and, thus, modeling psychometrics upon quantitative physics, as done since Spearman (1904) would be a false lead. Scientific progress requires conceptualizing relevant phenomena correctly. Were abilities, for example, heterogeneous orders, conceptualizing them as purely homogeneous attributes would blind investigators to distinctions between the degrees of any given ability and, so, the character of such attributes would be misunderstood. Just as understanding the workings of the human body requires first getting anatomical structures right, so understanding the workings of the human mind depends upon first getting psychological structures right.

Second, it would mean that the features of people assessed by psychological tests are best described, not numerically but qualitatively via a specification of, say, the knowledge, skills, and strategies displayed in getting ability test items correct. That is, for example, in testing mathematical ability, the optimal form for describing a person’s performance is not numerical (say, person *X* got 20 out of 30 correct answers or *X*’s ability “measure” is 7.5) but something like *X* knows *this* or *that* mathematical fact, or *X* can perform *this* or *that* operation or *X* can employ *this* or *that* solution strategy. In science, description needs to fit the structure of the attributes described and if abilities, etc., are heterogeneous orders, then qualitative description will be less misleading than quantitative.

Third, because the different degrees of any ability, say, would be qualitatively different, it would mean that people possessing different degrees would be subject to different causal laws. Then, for example, the kind of intervention that improves ability at one degree would not necessarily improve ability at other degrees. As already indicated, in quantitative sciences like physics, the lack of heterogeneity within each and every quantitative attribute sustains the system of homogeneous quantitative interrelationships that exist, such as force equals mass times acceleration. No such pattern of homogeneous laws could exist where the relevant attributes are not quantitative. If psychological attributes are heterogeneous orders, psychology will lack the simplicity characterizing quantitative physics. It will be a much more richly textured science, one in which the density of causal relationships will constantly challenge our cognitive capacities.

Fourth, if abilities, etc., are heterogeneous orders then it follows that psychometricians have misconstrued the problem of test validity. The concept of test validity is generally understood via the concept of “construct validity” (Cronbach and Meehl, 1955). The “construct” that a test is thought to “measure” is conceived as a theoretical, psychological, quantitative attribute of persons and, therefore, an attribute that is purely homogeneous, with no heterogeneous differences between degrees. However, it is significant that after 60 years, psychometricians have not yet managed to define a single psychological construct in terms of intrinsic characteristics. Constructs are generally defined as dispositional concepts by reference to the behaviors that are thought to cause, such as mathematical ability, which causes mathematical behavior; verbal ability, which causes verbal behavior, and so on. Furthermore, it is not made clear how a purely homogeneous attribute could sustain the heterogeneous differences between the cognitive states necessary for correct responses. On the other hand, if abilities are heterogeneous orders, there would be no longer any mystery about the character of the attribute assessed nor any mystery about how such an attribute produces correct responses (Michell, in press). In this case, what any ability item assesses would be just the knowledge, skills, and strategies required to get it correct, a cognitive state implied by the content of the item itself. That is, the issue of test validity would be exposed as an artifact of attempting to construe abilities as theoretical quantitative attributes. While intelligence or general ability is often thought of as a cognitive factor present to some degree in all intellectual tasks (such as Spearman’s “education of correlates”; Spearman, 1923, p. 284), no one knows whether there is any general property of our cognitive processes that contributes to individual differences in performance on all intellectual tasks and if the attribute assessed by any test is a heterogeneous order then there is no reason at this stage to conclude that any candidate for general ability must be quantitative in structure.

Fifthly, the fact that psychometricians, from the founding of their discipline, studiously turned away from investigating whether the attributes they aspired to measure really are quantitative means that their discipline is a pathological science (Michell, 2000) and that their standing as scientists is deeply compromised. Scientists who care more about appearing to be quantitative and the advantages that might accrue from that appearance, than they do about investigating fundamental scientific issues, put expedience before the truth. In this, they do not conform to the values of science and elevate non-scientific interests over those values, thereby threatening to bring science as a whole into disrepute. If the attributes that psychometricians aspire to measure are heterogeneous orders then psychometrics, as it exists at present, is fatally flawed and destined to join astrology, alchemy, and phrenology in the dustbin of history.

While this paper has been concerned primarily with historical issues, the matters discussed are not just historical. Raking over the coals of this episode, I have resurrected the concept of a heterogeneous order. Now, psychometricians have no excuse not to reconsider the structure of the attributes, which, hitherto, they concluded were quantitative. Considering the quotation from Binet and Simon with which this paper began, it could be argued that, properly understood, it says all that any non-psychometrician needs to know about psychological testing: testing may not be measurement, in the scientific sense, because the psychological states subtending performance on tests may not be quantitatively structured; such states might form merely ordered hierarchies of abilities, etc., characterized by heterogeneous differences between their degrees; but for all of the practical purposes to which test scores are currently put, since only ordinal information is used, it serves as well as actual measurements would, were they possible. But rather than follow Binet in therefore calling test scores “measurements,” it would be sufficient for all scientific purposes to call them merely “assessments” and we must look for other, non-scientific reasons should we wish to understand why psychometricians have not always adopted this more modest, accurate appellation. As for measurement, the burden of proof now lies with psychometricians for even with the best of tests the default position now is that the attributes assessed are merely heterogeneous orders.

## Conflict of Interest Statement

The author declares that the research was conducted in the absence of any commercial or financial relationships that could be construed as a potential conflict of interest.

## Footnotes

^1^For the concept of scientific measurement see Michell (1997, 2003, 2007).

^2^Alfred Binet (1857–1911).

^3^Lewis Madison Terman (1877–1956).

^4^The BinetSimon scale was first published in 1905 and revised in 1908 and 1911.

^5^The first Stanford–Binet scale was produced in 1916 and went through numerous revisions.

^6^Binet and Simon (1905). Wolf (1961) notes that this 1905 paper was actually written solely by Binet, as were most of their nominally co-authored papers. Hence, I refer to Binet as if the sole author.

^7^The 1980 edition of an English translation of seminal papers by Binet and Simon, from which the above passage is quoted, is a re-issue of a 1916 edition of the same translation containing Terman’s marginalia.

^8^Jules Tannery (1848–1910). Tannery received his doctorate from the École Normale Supérieur in 1874 and later was professor of mathematics there. His critique of Fechner, made just after his graduation, is in Tannery (1875a,b) and reprinted in Tannery (1912). No English translation is yet published. I am grateful to Christian Bethmont for translating it for me.

^9^The epistemological realist would argue that sensations are not measurable because there are no such things as sensations. There are physical quantities, such as temperature and there are features of such quantities, such as order and ratio, that humans may or may not be sensitive to under various conditions, but there are no mental entities, *sensations*, as both Fechner and Tannery believed. In this paper, however, I am not concerned with this issue, but with the fact that his belief in sensations was the vehicle whereby Tannery introduced the concept of a heterogeneous order. So as not to deflect attention from this, my discussion maintains the fiction that sensations exist.

^10^This argument is often referred to as the “quantity objection” to psychophysical measurement. A similar, but better-known version of this objection was presented later by von Kries (1882).

^11^Henri Bergson (1859–1941) was the most important French philosopher of the first half of the twentieth century. An English translation of his critique of psychophysics (Bergson, 1889) is given in Bergson (1913). Interestingly, it was Bergson who was responsible for thwarting Binet’s career aspirations (Nicolas and Ferrand, 2002).

^12^The argument from order to quantity I call “the psychometricians’ fallacy” (Michell, 2009, 2012) because (a) it is a demonstrable logical fallacy and (b) it played a fundamental role in psychometrics.

^13^This was the position later adopted in psychometrics, where it is held that attributes are either *categories* or *continua*, e.g., Cronbach and Meehl (1955), Loevinger (1957), Meehl (1992), and De Boeck et al. (2005). Bergson should be recognized as the “patron sage” of psychometrics.

^14^For example, note the place it occupies in Titchener (1905).

^15^For example, in his book on the mind-body problem, Binet (1905) criticized Bergson extensively. Binet appears not to have thought too highly of the man standing in his way.

^16^This paper is discussed in Carroy and Plas (1996).

^17^Usage is not completely standard in this context. I use the term “magnitude” to refer to specific instances of a quantitative attribute, such as length. Thus, the length of my shoe, which may be, say, 30 cm, is a magnitude of length.

^18^Or at least this was De Morgan (1836) interpretation of him.

^19^See Plato’s (1993) *Philebus*.

^20^See Aristotle’s *On Generation and Corruption* (McKeon, 1941).

^21^The idea of “degrees of a quality” should not be confused with the concept of “degree” as it occurs in, say, the measurement of angle or temperature. In the former, the term “degree” implies nothing more than order, while in the latter it designates a unit of measurement.

^22^*Categories*, 6^a^ 19 (McKeon, 1941, p. 17).

^23^*Categories*, 10^b^ 26 (McKeon, 1941, p. 27).

^24^John Duns Scotus (1266–1308). For a discussion of Duns Scotus’ suggestion, see Cross (1998).

^25^Reid (1748/1849, p. 717). Reid was attacking Hutcheson’s (1725) proposed moral algebra.

^26^Ducasse (1941, p. 42). Ducasse was criticizing Collingwood’s (1933) concept of a “scale of forms,” which was that of a heterogeneous order by another name.

^27^See Sutherland’s (2004) discussion of Kant’s views.

^28^Binet’s book on the mind/body problem paid attention to Kant’s views (Binet, 1905), but whether he knew of Kant’s views on the philosophy of measurement I do not know.

^29^See Hölder (1901) and Krantz et al. (1971). Krantz et al. show that an attribute’s being quantitative is equivalent to a special kind of ordering upon the differences between magnitudes.

^30^For example, Black et al. (2011), Commons et al. (2008), Kyngdon (2006), and Kyngdon and Richards (2007) all present tests in which the only discernable structure manifest in the item sets is ordinal and yet IRT models fit the relevant data. I thank Andrew Kyngdon for drawing my attention to the first two of these references.
